# Immune dysregulation associated with co-occurring germline *CBL* and *SH2B3* variants

**DOI:** 10.1186/s40246-022-00414-y

**Published:** 2022-09-19

**Authors:** Francesco Baccelli, Davide Leardini, Edoardo Muratore, Daria Messelodi, Salvatore Nicola Bertuccio, Maria Chiriaco, Caterina Cancrini, Francesca Conti, Fausto Castagnetti, Lucia Pedace, Andrea Pession, Ayami Yoshimi, Charlotte Niemeyer, Marco Tartaglia, Franco Locatelli, Riccardo Masetti

**Affiliations:** 1 Pediatric Oncology and Hematology “Lalla Seràgnoli”, IRCCS Azienda Ospedaliero-Universitaria Di Bologna, 40138 Bologna, Italy; 2 Department of Medical and Surgical Sciences (DIMEC), University of Bologna, 40138 Bologna, Italy; 3 Chair of Pediatrics, Department of Systems Medicine, University of Rome Tor Vergata, 00133 Rome, Italy; 4 Immune and Infectious Diseases Division, Research Unit of Primary Immunodeficiencies, Academic Department of Pediatrics, IRCCS Ospedale Pediatrico Bambino Gesù, Rome, 00165 Rome, Italy; 5 Pediatric Unit, IRCCS Azienda Ospedaliero-Universitaria Di Bologna, 40138 Bologna, Italy; 6 Hematology “Lorenzo E Ariosto Seràgnoli”, IRCCS Azienda Ospedaliero-Universitaria Di Bologna, 40138 Bologna, Italy; 7 Department of Experimental, Diagnostic and Specialty Medicine (DIMES), University of Bologna, 40138 Bologna, Italy; 8 Department of Hematology/Oncology and Cell and Gene Therapy, IRCCS Ospedale Pediatrico Bambino Gesù, Catholic University of the Sacred Heart, Rome, 00165 Rome, Italy; 9 Division of Pediatric Hematology and Oncology, Department of Pediatrics and Adolescent Medicine, Medical Centre, Faculty of Medicine, University of Freiburg, 79085 Freiburg, Germany; 10 Genetics and Rare Diseases Research Division, IRCCS Ospedale Pediatrico Bambino Gesù, 00165 Rome, Italy

**Keywords:** CBL, JMML, Immune dysregulation, CBL syndrome, SH2B3

## Abstract

**Background:**

CBL syndrome is a RASopathy caused by heterozygous germline mutations of the Casitas B-lineage lymphoma (CBL) gene. It is characterized by heterogeneous clinical phenotype, including developmental delay, facial dysmorphisms, cardiovascular malformations and an increased risk of cancer development, particularly juvenile myelomonocytic leukemia (JMML). Although the clinical phenotype has been progressively defined in recent years, immunological manifestations have not been well elucidated to date.

**Methods:**

We studied the genetic, immunological, coagulative, and clinical profile of a family with CBL syndrome that came to our observation after the diagnosis of JMML, with homozygous CBL mutation, in one of the members.

**Results:**

Variant analysis revealed the co-occurrence of CBL heterozygous mutation (c.1141 T > *C*) and *SH2B3* mutation (c.1697G > *A*) in two other members. Patients carrying both mutations showed an ALPS-like phenotype characterized by lymphoproliferation, cytopenia, increased double-negative T-cells, impaired Fas-mediated lymphocyte apoptosis, altered cell death in PBMC and low TRECs expression. A coagulative work-up was also performed and showed the presence of subclinical coagulative alterations in patients carrying both mutations.

**Conclusion:**

In the reported family, we described immune dysregulation, as part of the clinical spectrum of CBL mutation with the co-occurrence of SH2B3.

**Supplementary Information:**

The online version contains supplementary material available at 10.1186/s40246-022-00414-y.

## Introduction

CBL syndrome [MIM 613563] is a RASopathy caused by heterozygous germline variants of the Casitas B-lineage lymphoma (*CBL*) gene [MIM 165360] [1]. *CBL* encodes an E3 ubiquitin-protein ligase that negatively regulates intracellular signaling, including the RAS-MAPK pathway [2]. Pathogenic *CBL* variants are predominantly missense or splice site changes affecting the RING finger domain and linker region and resulting in defective ubiquitin ligase activity. The clinical phenotype of CBL syndrome is highly variable and overlaps with Noonan syndrome (NS) [OMIM 163,950, PS163950] [3, 4], the most common disorder among the RASopathies [5]. Although the clinical manifestations have progressively been defined in recent years [3], the complete clinical spectrum seems far to be entirely described. Impaired growth, cognitive deficits, cryptorchidism, distinctive facial features, cardiovascular malformations and vasculitis have been reported [6]. Patients with RASopathies are known to be at increased risk of cancer development, CBL syndrome is associated with an increased risk of JMML and *CBL* mutations occurs in of a variety of myeloid neoplasms [7, 8]. Leukemogenesis in germ-line mutations is mainly related to the loss of wild-type *CBL* allele in somatic hematopoietic stem cells (HSCs) by acquired uniparental isodisomy of the 11q23 chromosomal region encompassing the mutated allele [7, 9]. Higher incidence of vasculitis and other autoimmune phenomena (e.g., cytopenia, uveitis) have also been described in patients with CBL syndrome [6, 10–12]. The pathogenesis of these autoimmune manifestations may be related to an acquired resistance to death signals in *T*-cell maturation process due to the inactivation of E3 ubiquitin ligase activity of CBL [13]. It has been shown that mice lacking *CBL* in *T* and *B* cells develop severe vascular lesions with massive infiltration of *T* cells and a lupus-like syndrome associated with perivascular infiltration and hyperactivation of B-cell receptor (BCR) signaling [14, 15]. While the biological role of *CBL* in immune homeostasis has been partially elucidated [16], specific immunological features of patients with CBL syndrome have not accurately been characterized so far. Lymphocyte adaptor protein (LNK), also called SH2B3 [MIM 605093], as referred hereafter, is a member of the Src homology 2 (SH2) domain-containing family of adaptor proteins, involved in modulating cytokines and growth hormone signaling by JAK/STAT pathway modulation. It has been described as a regulator of hematopoiesis and lymphocyte differentiation [17]. SH2B3 negatively regulates intracellular signaling elicited by different cell-surface receptors, such as c-kit, thrombopoietin receptor and erythropoietin receptor, all converging towards the activation of the JAK-STAT cascade [18]. Somatic mutations in *SH2B3* gene have been described in myeloproliferative neoplasms [19], while germline *SH2B3* pathogenic variants have been reported in myelodysplastic syndromes and acute lymphoblastic leukemia [20, 21]. Variants in *SH2B3* were also described in autoimmune diseases, particularly autoimmune type 1 diabetes [22]. CBL and SH2B3 are functionally related, contributing to the modulation of signal strength promoted by JAK proteins [23]. We herein report a family segregating pathogenic variants in *CBL* and *SH2B3* presenting novel clinical features, particularly related to immune dysregulation.

## Material and methods

### Genetic analysis

**Fig. 1:**
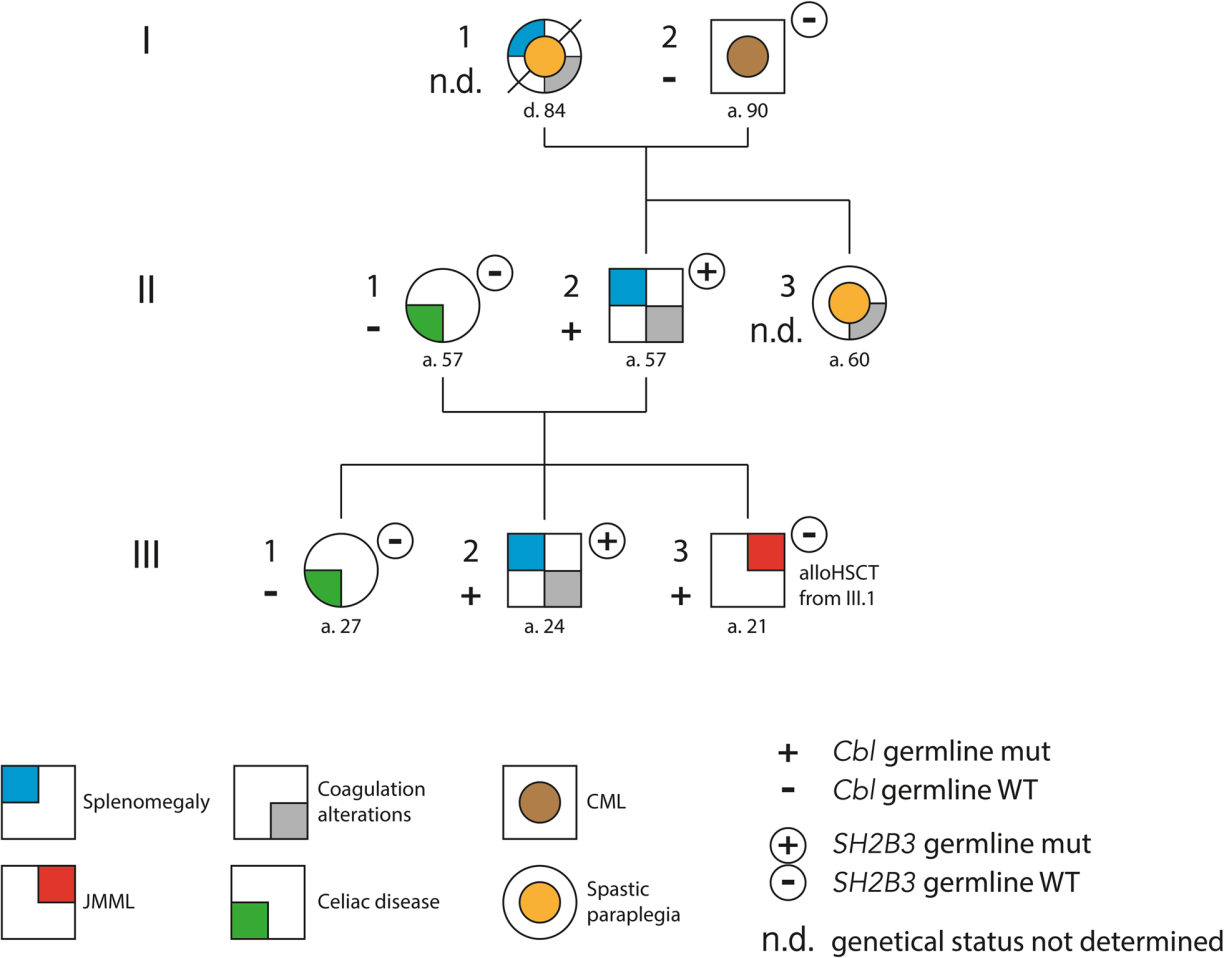
Pedigree of the described Italian family. Figure summarizes clinical and genetic characteristics of the reported family. Patient I.2 is affected by Philadelphia positive chronic myeloid leukemia and is receiving treatment with Imatinib. The analysis of CBL variants performed on peripheral blood during clinical remission resulted negative. CML: chronic myelogenous leukemia; HSCT: hematopoietic stem cell transplantation; JMML: juvenile myelomonocytic leukemia

DNA from peripheral venous blood was available from subjects: I.2, II.1, II.2, III.1, and III.2. In addition, DNA specimens from peripheral blood mononuclear cell (PBMC) before transplantation and buccal mucosa after transplantation were available for subject III: 3 (Fig. 1). Following purification (QIAamp DNA Mini kit, Qiagen), genomic DNA was used to assess the mutational profile of the affected individuals by capture-based parallel sequencing using a NextSeq550 platform (Illumina). A total of 554 genes were included in the designed gene panel (list available on request) using custom-designed NimbleGen SeqCap probe hybridization (Roche NimbleGen).Read alignment to the reference genome (UCSC-GRCh37/hg19) and variant calling, filtering and prioritization analyses were performed using an in-house implemented pipeline mainly based on the GATK Best Practices (GATKv3.7, http://www.broadinstitute.org/gatk/). Variants of possible clinical interest were extracted by setting filtering criteria on population frequency, as well as disease associations and in silico prediction of impact.

Segregation analyses were performed by Sanger sequencing. PCR primers on the *CBL* and *SH2B3* sequence have been drawn with the Primer Express software in the regions flanking *CBL* exon 8 (F: 5’-GGACCCAGACTAGATGCTTTCT-3’ and R: 5’-TCGCTGTTTAGATCCGTACCTG-3’) and *SH2B3* exon 7 (F: 5’-AGGTCTGACCCTACTGCCC-3’ and R: 5’-GCCAGGTGGATAGATGAAACCT-3’).

PCR was performed using the FastStart Taq DNA Polymerase kit (Roche) according to the manufacturer instruction with the following program: 95 °C for 5 min, (95 °C for 30 s, 60 °C for 30 s, 72 °C for 30 s) for 35 cycles, 72 °C for 7 min. Samples were sequenced using the BigDye Terminator v1.1 Cycle Sequencing kit (Applied Biosystems) and loaded on the 96-capillary ABI 3730 DNA Analyzer (Applied Biosystems), and sequences were read using the Sequencher 4.1 software (Gene Codes Corporation).

### Immunological analysis

#### Immunophenotype by flow cytometric analysis

On peripheral blood, after red blood cell lysis with ammonium chloride, the following antibodies were employed: CD3 PerCP (clone BW264/56, MiltenyiBiotec, BergischGladbach, Germany), CD4 APC (clone OKT4, Becton Dickinson, Franklin Lakes, NJ, USA), CD8 PE-Cy7 (clone RPA-T8, Becton Dickinson, USA), TCR alpha–beta APC (clone T10B9, Becton Dickinson), TCR gamma-delta FITC (11F3, MiltenyiBiotec, DE), CD45RA APC-H7 (clone T6D11, MiltenyiBiotec, DE), CCR7 PE (clone 3D12, Ebioscience, San Diego, CA, USA), CD127 PE-CY7 (clone eBioRDR5, eBiosciences), CD16 PE (clone 3G8), CD56 PE (clone NCAM16.2), CD19 PE-CY7 (clone SJ25C1, Becton Dickinson), CD27 FITC (clone M-T271, Becton Dickinson), CXCR5 (clone J252D4 Biolegend), CD31 (cloneWM59, Miltenyi Biotec, DE).

#### ELISA Immunoassay for IgM, IgA, and IgG

Plasma and secreted Igs were quantified with ELISA immunoassay after stimulation with CpG. 96-well plates (Corning) were coated with purified anti-human IgA, IgG, and IgM (Jackson ImmunoResearch Laboratories, PA, USA), washed with PBS/0.1% Tween and blocked with PBS/1% gelatine. Subsequently, two incubation steps for 1 h at 37 °C, first with culture supernatants and second with peroxidase-conjugated goat anti-human IgA, IgG, or IgM Abs (JacksonImmunoResearch Laboratories, PA, USA) were performed. Chromogenic substrate was *o*-phenylene-diamine solution (Sigma-Aldrich, St. Louis, MO, USA).

#### Lymphocyte proliferation assay

PBMC were labeled with 5 µM of carboxyfluorescein diacetate succinimidyl ester (CFDA-SE) [8 min at room temperature]. Plates of CFSE-stained cells were stimulated for 72 h with antiCD3/CD28 beads (Dynabeads Human T-activator CD3/CD28, Life Technologies, 50 beads/1 CD3) or PHA (10 μg/mL, Roche). Proliferating T cells were stained with anti-CD4 PeCy7, 7-Amino-actinomycin D (7-AAD) and anti-CD8 APC. Final analysis was performed with FlowJo software 2.2 (TreeStar Inc, Ashland, Ore).

#### Fas-mediated apoptosis test

Fas-mediated apoptosis was tested on T-cells. PBMCs were activated with phytohemagglutinin (PHA) at days 0 (1 mg/mL) and 15 (0.2 mg/mL) and cultured in RPMI 1640 plus 10% fetal calf serum plus recombinant interleukin-2 (IL-2; 5 U/mL; Biogen, Geneva, Switzerland). On day 21, cells were incubated with anti-Fas MoAb (IgM isotype; 1 mg/mL; UBI, Lake Placid, NY) or anti-Fas MoAb plus anti-IgM rabbit antimouse serum (1 mg/mL; Serotec, Oxford, UK). IL-2 (5 U/mL) was added to minimize spontaneous cell death. Cell survival was evaluated after 18 and 48 h. Surviving cells were counted by trypan blue exclusion test. The results were expressed as percentage of specific cell survival.

#### TRECs expression

Real-time quantitative PCR for TRECs was performed by a ViiA7 real-time PCR system (Applied Biosystems, Foster City, Calif) and optimized based on custom reagents provided by Affymetrix (Santa Clara, Calif). TREC levels were normalized per microliter of blood, considering that single 3.2-mm punch contains approximately 3 μL of whole blood. Abnormal TREC results were defined as less than 8 TRECs/μL of dried blood.

#### Phospho-S6 protein assay

Ex vivo assessment of S6 Ser235/236 phosphorylation was performed on whole blood using an antibody against phosphorylated S6 Ser235/236 (clone D57.2.2E, catalog 8520S, Cell Signaling Technology) and the PerFix EXPOSE kit, according to the manufacturer’s recommendations (Beckman Coulter). Briefly, 100 μl of fresh blood was incubated in the presence of surface antibody for 10 min at 37 °C in a water bath. B- and T-cell surface markers were: CD19 (clone HIB19, catalog 2,111,030, Sony), CD3 (clone UCHT1, catalog 25-0038-42, BD), CD4 (clone VIT4, catalog 130-092- 373, MiltenyiBiotec), CD8 (clone BW135/80, catalog 130-096-902, MiltenyiBiotec). The reaction was stopped using buffer 1 and red blood cells were lysed with buffer 2 for 5 min at 37 °C. The samples were centrifuged, and the pellets underwent intracellular staining with buffer 3 for 1 h. The cells were then washed with the dedicated buffer, re-suspended, and analyzed with a FacsAriaIII flow cytometer (Becton–Dickinson).

#### Patient consent and ethical approval

The patients gave written informed consent for the clinical evaluations and genetic analyses prior to inclusion in the study, in accordance with the ethical standards of the institutional research committee and with the 1964 Helsinki declaration and its later amendments or comparable ethical standards. All the analyses were performed within the common clinical practice of the treating hospital, the IRCCS Azienda Ospedaliero-Universitaria di Bologna, and thus no specific research protocol was approved in accordance with the Area Vasta Emilia Centro Ethic Committee requirements. The clinical data for the patients were obtained from questionnaires completed by the attending physician, as well as from medical records. Informed consent was obtained from the patient to publish the images in an online open access publication.

## Results

### III.3 presented CBL syndrome and developed JMML due to a second somatically acquired hit.

Figure 1 summarizes selected clinical, pathologic, and molecular findings of the analyzed family. The proband (III.3) came to our attention in 2000 for the presentation of severe anemia and thrombocytopenia associated with marked splenomegaly during infancy. Diagnosis of Juvenile Myelomonocytic Leukemia (JMML) was made according to clinical features, laboratory criteria and hypersensitivity to GM-CSF [24]. For the subsequent worsening of the clinical conditions and severe transfusion dependency, the patient received allogeneic HSCT from a fully HLA matched sibling donor (III.1). Conditioning regimen with Busulfan, Thiotepa and Cyclophosphamide and standard cyclosporine-based graft-vs-host disease (GvHD) prophylaxis were used, and 8.73 × 108 total nucleated bone marrow cells/kg were infused. He did not present major early post-HSCT complications, and currently shows complete donor chimerism. Three years after HSCT, he developed hypothyroidism. During the 21 years of follow-up, he never showed recurrence of the primary disease. During his late adolescence, he developed pericardial effusion that resolved spontaneously. He presented with typical features of CBL syndrome, including facial dysmorphisms (Additional file 1: Fig. S1), and mild cognitive impairment. He also showed dental agenesis, tracheomalacia, multiple lipomas and cerebellar tonsils herniation. Considering this clinical phenotype alongside with the previous diagnosis of JMML, we suspected CBL syndrome. Genetic analysis performed on DNA from buccal mucosa and hair follicles showed a heterozygous variant at chromosomal position chr11:119,148,921 (GRCh37/hg19) in *CBL* (c.1141 T > C; GenBank: NM_005188.4). The nucleotide change affected exon 8 of the gene, and predicted the substitution of a cysteine residue at codon 381 by arginine [p.Cys381Arg]. Cys381 is a highly conserved residue located in the RING finger domain, whose proper function is required for the ubiquitin ligase activity of CBL. In silico predictive algorithms consistently indicated a deleterious effect on protein structure/function. This variant has been annotated in the dbSNP database (rs757874631), but it was not reported in gnomAD (Version: 2.1). The variant was classified as pathogenic according to the recommendation of American College of Medical Genetics and Genomics (ACMG), and was reported as pathogenic in the COSMIC database (COSM34056); however, conflict interpretations of pathogenicity (i.e., likely pathogenic vs variant of uncertain significance [VUS])were reported in the ClinVAr database. Following analyses on stored bone marrow samples collected after JMML diagnosis and before HSCT documented homozygosity for the *CBL* variant. The somatically acquired LOH resulted from isodisomy.

### III.2 and II.2 showed CBL syndrome with ALPS-like features and harbored a concurrent missense variant in the SH2B3 gene

This finding prompted us to extend genetic testing in family members, revealing the occurrence of the same *CBL* variant in individuals III.2 and II.2 (Fig. 1). The variant was absent in DNA samples extracted from peripheral blood and buccal swab of the paternal grandfather (I.1). The lack of paternal grandmother’s samples did not allow us to conclude whether it was a de novo or an inherited event. Like his younger brother, subject III.2 presented with mild facial dysmorphism and mild cognitive impairment. Additional findings include mixed bilateral hearing loss, dermatofibroma, brachydactyly, inguinal hernia and horseshoe kidney. In the first years of life, the subject suffered from recurrent petechiae and easy bruising. During his early childhood, he was referred to our tertiary center for splenomegaly, micro-hematuria, and mild thrombocytopenia (platelet count, 87.000/microliter) in presence of anti-platelet antibodies. In the suspicion of autoimmune lymphoproliferative syndrome (ALPS), Fas-mediated lymphocyte apoptosis assay was performed and resulted altered (95% of surviving lymphocytes, normal values [n. v.] < 82%). Increased double negative T (DNT) cells CD3 + CD4-CD8-TCR α/β + (3.10% of CD3 + , n. v. < 2.5%), leading to the diagnosis of ALPS according to the revised criteria from the 2009 NIH International Group [25]. Genetic analysis excluded pathogenic variants in *FAS*, *FASL* or *CASP10* and the disease was classified as ALPS-U (unknown genetic cause) [25]. During his middle childhood, he showed mild anemia (Hb, 12.1 g/dL), leukopenia (white blood cells, 580/microliter), thrombocytopenia (platelet count, 103.000/microliter) and persistent splenomegaly. Morphological evaluation of bone marrow (BM) smear revealed hypercellularity with mild-to-moderate left shift in myeloid maturation, pelgeroid granulocytes and absence of dysmorphic features (Additional file 1: Fig. S2). Mild dysmorphism was also noticed in subject II.2. ALPS was diagnosed due to the presence of chronic splenomegaly, together with defective Fas-mediated lymphocyte apoptosis (83% of surviving lymphocytes) and increased DNT (2.9% of CD3 +), while complete blood count resulted normal. Of note, vitamin B12 levels were within normal limits both in patient II.2 and in patient III.2. Targeted sequencing allowed to identify a missense variant in the *SH2B3* gene (c.1697G > A; GenBank: NM_005475.3) in both individuals (Fig. 1; Additional file 1: Fig. S3). The variant was absent in individuals I.2, II.1, III.1, and III.3. We cannot conclude whether it was a de novo or an inherited event in II.2. The missense change is predicted to replace an arginine residue at codon 566 by glutamine (p.Arg566Gln). This variant was classified as a VUS according to the ACMG guidelines and had previously been reported in the dbSNP database (rs148791142), but not in ClinVar database, and has an extremely low frequency in gnomAD (ƒ = 0.0000878; Version:2.1). It was reported as pathogenic in COSMIC (COSM6947187). Consistent with the molecular findings, subjects I.2, II.1 and III.1 did not show any constitutional characteristics and pathological features resembling CBL syndrome. Of note, subject I.2 was affected by a Philadelphia positive chronic myeloid leukemia in treatment with Imatinib. Subject II.3 presented type 4 spastic paraplegia with an intragenic heterozygous deletion of the *SPAST* gene (2p22.3), anti-phospholipid syndrome with suspected neurological involvement, bilateral retinal detachment, endometriosis, recurrent oral ulceration, alopecia, and splenomegaly. Laboratory findings included positive antinuclear antibody, Lupus anticoagulant (LAC) and anti-platelets antibodies in the absence of thrombocytopenia, together with non-specific alterations at the capillaroscopy. However, diagnostic criteria for systemic lupus erythematosus (SLE) were not matched. Spastic tetraparesis involving the I and II motoneurons was also diagnosed in subject I.1, during the adult age, without a genetic characterization. Splenomegaly was also present in this subject. While the clinical features were suggestive of CBL syndrome in subjects II.3 and I.1, *CBL* genetic status is not available.

### Affected subjects show impaired cell death in PBMC and low TRECs expression in absence of PI3K-AKT pathway upregulation

To identify occurrence of immunological alterations associated with the *CBL* and *SH2B3* variants, extensive immunological characterization was carried out (Table 1, Additional file 1: Table S1).

**Table 1:** Results of immunological characterization of the studied family. DNT, γ + δ + T and effector CD8 + memory cells are increased in II.2, III.2 and III.3; II.2 shows a B-cell memory deficiency

	II.1	II.2	III.1	III.2	III.3 (I-135)	Normal values for age (> 16 yrs.)
*Immunophenotyping of peripheral blood cells*						
WBC 10^9 /L	6.54	5.24	5.41	7.01	3.85	3.60–10.50
Lymphocytes 10^9 /L	1.92	0.81	2.07	2.05	1.51	1.2–4-1
Monocytes 10^9 /L	0.40	0.46	0.42	0.42	0.35	0.10–0.90
CD3 + (PAN T), % (cells/μL)	72%	65.1%	66%	73.3%	38.7%	50–91% lymph
CD3 + TCR α + β + , %	96%	87.5%	95%	89.3%	90.4%	36–98% CD3 +
CD3 + TCR γ + δ + , %	4%	12.3%	5%	10.3%	8.6%	0.83–11% CD3 +
CD3 + CD4-CD8-, (DNT) **%**	0.7%	2.9%	2.7%	2.4%	2.6%	0.57–3% CD3 +
CD3 + CD4, % (cells/μL)	43% (825)	42.6%	33% (683)	27.4% (561)	19.8% (298)	28–64% CD3 + (500–2000)
CD4 + CD45 RA + (Naïve), %	21%	23.3%	50%	48.5%	37%	16–100% CD4 +
CD4 + CD45 RA-CCR7 + (Central memory), %	55%	70%	42%	48%	56.2%	18–95% CD4 +
CD4 + CD45 RA-CCR7- (Effector memory), %	20%	6.6%	10%	3.4%	6.8%	1–23% CD4 +
CD4 + CD45 RA + CCR7- (Terminal effector memory), %	3%	0.1%	< 0.1%	0.08%	0.08%	0.0083–6.8% CD4 +
CD4 + CD127 + CCR7 + CD25 + + (T reg)	4	n.d	4	n.d	n.d	4–17% CD3 +
CD3 + CD8, % (cells/μL)	25% (480)	10.8%	26% (538)	39.6% (811)	16% (241)	12–40% CD3 + (200–1200)
CD8 + CD45 RA + (Naïve), %	22%	11.6%	39%	11.2%	20.8%	6–100% CD8 +
CD8 + CD45 RA-CCR7 + (Central memory), %	24%	2.8%	10%	2.0%	2.2%	1–20% CD8 +
CD8 + CD45 RA-CCR7- (Effector memory), %	25%	79.8%	34%	78.3%	68.7%	14–98% CD8 +
CD8 + CD45 RA + CCR7- (Terminal effector memory), %	28%	5.6%	18%	8.5%	8.4%	7–53% CD8 +
CD3-CD56 + CD16 + (NK), %	17%	25%	21%	12.7%	44.8%	5–49% WBC
CD3 + CD56 + CD16 + (NKT), %	n.d	4.2%	n.d	3.8%	1.7%	1–18% WBC
CD19 + (PAN B), % (cells/μL)	10.66% (203)	5.2%	11.7% (242)	8.7% (178)	14.4% (217)	4–28% (64–820) lymph
CD19 + IGD + CD27- (B naïve)	45%	47.7%	59%	86.70%	72.40%	33–100% CD19 +
CD19 + IgD + CD27 + (unswitched B memory)	12%	25.10%	21%	1.70%	6%	3–61% CD19 +
CD19 + IgD-CD27 + (switched B memory)	33%	29.70	14%	11.60%	14%	3–46% CD19 +
CD19 + CD21 + CD38- (B CD21 + low)	7%	4.4%	7%	1.1%	2.6%	2–14% CD19 +
CD19 + IgM + + CD38 + + (B transitional)	3.6%	2.3%	1%	9%	7.1%	0.27–24 CD19 +
CD19 + IgM- + CD38 + + (B plasmablasts)	0.3%	0.10%	0.3%	0.10%	0.02%	0.7–6% CD19 +
*Lymphocyte proliferation with anti-CD3/anti-CD28 + rec. IL2 and with PHA*						
Proliferating Lymphocytes with anti-CD3/anti-CD28 + rec. IL2 [%]	76	97	79	66	94	> 75%
Proliferating Lymphocytes with PHA [%]	92	96	98	95	96	> 75%
*Fas-mediated apoptosis test*						
Fas-mediated apoptosis (T lymphocyte survival, %) [< 82%]	64%	84; 83^1^	75%	95; 82^a^	n.d	< 82%
*TRECs expression, RTE, TFH*						
TRECs expression	Normal	Low	Normal	Low	Low	
CD4 + CD45RA + CD31 + (RTE) %	n.d	13.4	n.d	32	27.3	7–100%
CD4 + CD45R0 + CXCR5 + (TFH) %	n.d	13.7	n.d	4.8	9.6	5–56%
*PhosphoS6 + lymphocytes*						
CD3 + phospho S6 + , %	3.58	5.1	8.51	8.2	5.25	% Controls (*n* = 23) 7,3 (2,6–17,4%) median (10–90°pc)
CD19 + phosphoS6 + , %	13.1	5.1	15.8	5.8	13.2	% Controls (*n* = 23) 6,4 (2,2–26,4%) median (10–90°pc)

Fas-mediated apoptosis is defective in II.2 and III.2. Fas-mediated apoptosis is defective in II.2 and III.2. TRECs expression is reduced in II.2, III.2 and III.3.DNT: double negative T lymphocytes; pS6 + : phosphoS6 + ; PHA: Phytohemagglutinin; RTE: recent thymic emigrants; TFH: T follicular helper lymphocytes; TRECs: T-cell receptor excision circles

aThe test was repeated in two separate assays

*nd* not determined

**Fig. 2:**
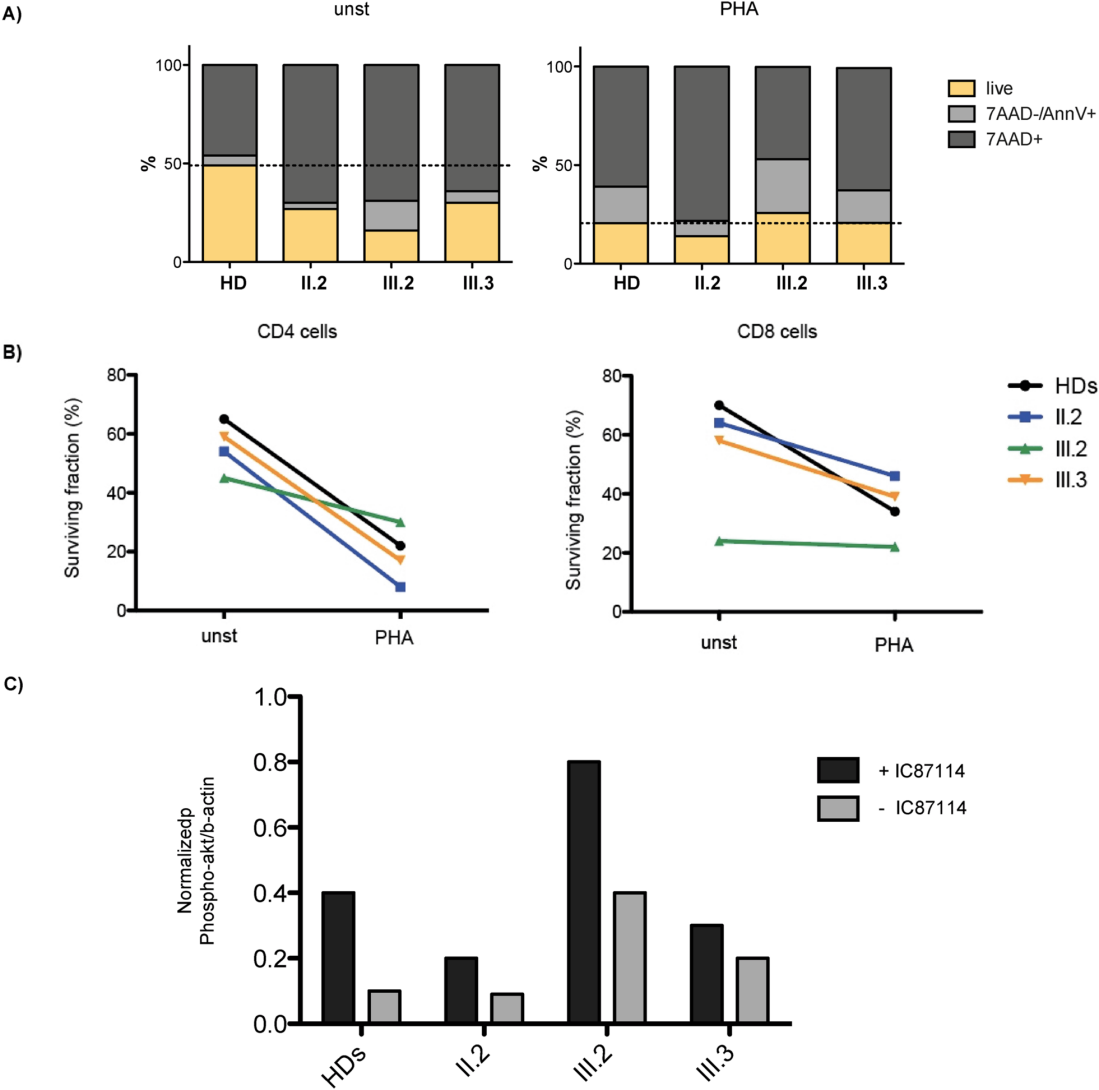
Immunological characterization. **A** Percentage of viable (7AAD-/AnnV-), apoptotic (7AAD-/AnnV +) or necrotic (7AAD +) PBMC investigated on patients II.2, III.2 and III.3 after 24 h of PHA-stimulation (1ug/ml); **B** surviving fraction (%) of CD4 and CD8 cells with or without PHA-stimulation; **C** Modulation of pAKT expression by IC87114 inhibitor (5 mM, for 30’) performed graphed as ratio between expression of pAKT /expression of B-actin (WB)

Peripheral blood immunophenotyping by flow-cytometry analysis showed increased DNT in patients with CBL syndrome. Of note, subject III.1 presented increased DNT levels, not presenting any genetic or pathological findings of CBL syndrome. The role of DNTs remains a matter of debate since they represent a shared feature for other non-ALPS autoimmune diseases, such as celiac disease, and thus cannot be considered a specific CBL syndrome feature [26–28]. Extensive immunophenotyping in patients with CBL syndrome highlighted other modest alterations in T-cell subsets, showing γδ + T-cell and effector CD8 + T memory cell expansion, suggesting that the immune CBL-mediated dysregulation may primarily affect T lymphocytes. No major alteration was noted in the B-cell population, presenting with normal immunoglobulin (Ig) levels and antibody responses in all tested members, with the exception of partial memory deficiency detected in subject II.2. Resistance to apoptosis via Fas/Fas ligand was analyzed, resulting defective in subjects II.2 and III.2 (data not shown), similar to what is typically described in ALPS [25]. Further studies were performed on peripheral blood mononuclear cells (PBMC) to investigate spontaneous or PHA-stimulated cell death, showing an increment of death in unstimulated patients’ cells, with any relevant differences with respect to healthy donors (HDs) in stimulated cells (Fig. 2A). Notably, evaluation of the surviving fraction of PHA-stimulated CD4 and CD8 subsets documented a mild resistance to cell death in CD8 cells from all three investigated patients (II.2, III.2 and III.3) and in CD4 cells from patient III.2 but revealed that CD4 cells from patients II.2 and III.3 have a cell death resistance comparable to that of HDs (Fig. 2B). These findings revealed an impaired cell death mechanism in patients’ cells. Subjects II.2, III.2, and III.3 showed low T-cell receptor excision circles (TRECs) expression compared to the other adult healthy family members (Table 1). Thymic output generally decreases with age, but an adequate output is still maintained into late adulthood. We noticed a significant difference between the patients and adult healthy controls. Considering the role of CBL in regulating phosphatidylinositol 3-kinase (PI3K)/AKT/mTOR pathway in mouse models [2, 13], we tested phosphoS6 basal levels on both T- and B-cell subsets, and AKT phosphorylation on PHA-stimulated T cells, as markers of PI3K activity. No significant differences were identified. Equivalent modulation of phospho-AAK by IC87114, an inhibitor of the pathway, was also observed, suggesting that *CBL* mutation does not alter the PI3K-AKT/mTOR signaling cascade (Fig. 2C).

### Mild coagulative alterations in patients affected by CBL syndrome

We then performed systematic revision of the clinical records to search for possible clinical signs of vasculopathies. We found that subject III.3 experienced an episode of pericarditis, occurred about 20 years after transplantation. The attribution of this pericarditis to CBL syndrome is controversial, since this clinical manifestation has not previously been reported in the disorder [23]. Moreover, subject III.3 received HSCT from the sister carrying wild-type *CBL* gene and a healthy immune system. We further performed a complete diagnostic work-up to reveal potential signs of endothelial alterations in the patient and other members of the family. Laboratory analysis revealed a pattern of hereditary coagulative alterations (Table 2).

**Table 2:** Results of coagulative analysis in the studied family

Patient	vWF reduction	LAC	INR (n.v. 0.9–1.2)	aPTT (n.v. 0.85 – 1.25)	Coagulation factors
III.1	–		Normal	Normal	n.d
III.2	+	–	1.22	1.50 (1.11 after correction with normal plasma)	Slight reduction in II, V, X, XI, XII
III.3 pre HSCT	–	+	1.54	1.48	n.d
III.3 post-HSCT	–	–	1.1	0.96	n.d
II.1	–	–	n.d	n.d	n.d
II.2	+	–	n.d	n.d	n.d
II.3	+	+	n.d	n.d	Slight reduction in fXI
I.1	+	+	n.d	n.d	n.d
I.2	–	–	n.d	n.d	n.d
I.3	+	+	n.d	n.d	n.d

III.2, III.2, II.3, I.1 and I.3 show a vWF reduction. Coagulation factors consumption was observed in III.2

Subject III.3 presented with positive LAC before HSCT and no alteration in the levels of von Willebrand factor (vWF) and D-dimer. However, prolongation of international normalized ratio (INR) and activated partial thromboplastin time (aPTT) was observed. Five years after HSCT from III.1, INR and aPTT of III.3 were normal, and LAC resulted negative. Slight reduction in the levels of vWF was observed in subject III.2, using both vWF antigen (0.56 UI/ml; n.v. 0.65–1.20) and vWF ristocetin cofactor (0.46 UI/ml; n.v. 0.70–1.50) alongside with a reduction in fVIII activity (0.55 UI/ml; n.v. 0.70–1.50). The patient’s blood group type was A negative. Prolongation of INR and aPTT was observed, with negative LAC. aPTT resulted normal after correction with normal plasma. Further coagulation work-up revealed slight reduction in the activity of fII, fV, fX, fXI, fXII, but no alteration in the levels of D-dimer was found. Subject II.2 received a diagnosis of a mild form of vW disease without clinical manifestations, and LAC was found to be negative. His sister, patient II.3, has asymptomatic vW disease, slight reduction in fXI and positive LAC. Finally, subject I.1 and her sister, identified as I.3, were both reported to be diagnosed with vW disease and to have positive LAC.

## Discussion

Here, we reported on a three-generation family transmitting a pathogenic *CBL* missense variant, which co-occurred with a likely pathogenic SH2B3 variant in two subjects. Besides the constitutional features fitting CBL syndrome, peculiar immunological alterations were documented in the two subjects heterozygous for the concomitant *CBL* and *SH2B3* variants.

Immune dysregulation has not extensively been described in patients with germline *CBL* mutations so far. However, CBL proteins (particularly CCBL and CBLB) have been demonstrated to regulate lymphocyte stimulation and the engagement of death-promoting signals by actively interacting with T-Cell receptor (TCR) signaling [2, 13]. Autoimmune and lymphoproliferative features observed in the present patients were initially diagnosed as manifestations of ALPS, but are likely to be related to CBL syndrome, particularly in the presence of a concomitant pathogenic variant in *SH2B3*. A comparable phenomenon has been associated with upregulated RAS function [29, 30]*.* Activating variants in genes coding for protein with role in RAS signaling are known to play a significant role in human diseases, including malignancies, and developmental and lymphoproliferative disorders [5, 31]. Activating mutations of *NRAS* and *KRAS* were also noted in a recently described pathological entity called ﻿RAS-associated autoimmune leukoproliferative disorder (RALD), which shares overlapping features with JMML and ALPS [32–34].

Germline mutations in *SH2B3* have been reported in different diseases, particularly myelodysplastic syndrome and JMML [19, 20, 35, 36]. Notably, SH2B3 was not mutated in our patient affected by JMML. Biallelic germline *SH2B3* mutations have been reported in two siblings with autoimmune manifestations [21]. While one of them developed a B-cell precursor ALL, the other presented a non-malignant clinical phenotype similar to II.2 and III.2 (immune cytopenia, developmental delay) [21]. Interestingly, association between type 1 diabetes and variants in the chromosome region 12q24 that contains SH2B3 has been demonstrated [37]. Occurrence of the *SH2B3* missense change was associated with higher T-cell proliferation on PMBCs, stimulated with CD28 and CD3, suggesting a role of altered SH2B3 function in the pathogenesis of autoimmune disorders [22].

Based on previous data indicating a role of c*-CBL* in TCR-induced thymocyte apoptosis in mouse models [13, 38], we investigated residual thymic output by measuring TRECs by real time quantitative PCR. In subject III.3, the reduced thymic function could be attributed at least in part to the toxic effect of HSCT. In addition, we hypothesize that thymic dysfunction could mainly be ascribed to the *CBL* deficiency, thus potentially contributing to the immunological alterations observed in the patients. We also speculate that even if HSCT effectively reconstituted a healthy immune system, germ-line *CBL* mutation in thymocytes could directly produce progressive thymus loss [38]. It should be noted that SH2B3 is expressed in hematopoietic progenitors, and his overexpression in lymphoid precursors impairs the expansion of T cells in thymus [39]. LNK/SH2B3 also negatively regulates IL-7-induced JAK/STAT signaling in B-cell progenitors [40]. Hematopoietic stem cells of Lnk deficient mice (Lnk -/-) presented with a high proliferative capacity resulting in splenomegaly and multi-lineage hyperplasia in BM together with an augmented ability to reconstitute the hematopoietic system in transplantation models [41–43].

Immune dysregulation could help to explain the vascular alteration and endothelial complications reported in CBL syndrome [6]. Some patients with JMML due to germline *CBL* mutations experiencing spontaneous resolution of the myeloproliferative/myelodysplastic disorder have been reported to develop different types of cardiovascular manifestations, including hypertension, cardiomyopathy, Moyamoya disease and Takayasu arteritis [6, 44]. Moyamoya disease has also been described in patients harboring de novo* CBL* mutations [10], and in CBL syndrome without JMML [45]. On the contrary, individuals with JMML and homozygous *CBL* mutations who undergo HSCT seem not to develop vasculitis, suggesting that a normal immune/hematopoietic system could be pivotal in preventing these manifestations [6]. Moreover, mice lacking *CBL* in peripheral immune cells present vascular infiltration of lymphocytes hypersensitive to TCR/BCR signaling, leading to endothelial damage [14, 15], highlighting the determinant role of immune dysregulation in vascular complications [6]. Recent emerging evidence also suggests a critical role of CBL in angiogenesis and endothelial cells signaling [16]. Defective CBL function seems to promote myofibroblast migration into the endothelium and subsequent proliferation via signaling elicited by the PDGF receptor, potentially contributing to the vascular disorders [46]. It is therefore reasonable that *CBL* mutations contribute to endothelial damage even if the underpinning of the biological mechanisms needs to be fully uncovered.

These findings collectively show the presence of a subclinical coagulative alteration with intrafamilial recurrence in the family members with CBL syndrome. Decreased vWF and coagulation factors consumption in these subjects could be attributable to diffuse activation of the coagulation system. We believe that these alterations are consistent with CBL syndrome, because they could be possibly related to endothelial damage due to CBL syndrome-associated vasculitis [6]. These features could be explained by increased platelet responses to GpVI agonists and thrombin linked with CBL loss of function, leading to increased platelet activation [47]. Interestingly, coagulative alterations have been reported in two patients with germline *CBL* mutations and Moyamoya disease [10]. The contribution of the co-occurring *SH2B3* variant to the coagulative alterations observed in subject II.2 and III.2 still needs to be fully characterized. SH2B3 has been shown to be a key negative regulator of signaling in endothelial cells, reducing activation and inflammation [48]. The normalization of the coagulation anomalies after allo-HSCT from his sister in subjects III.3 suggests a critical role of the hematopoietic and immune system in these alterations, rather than a direct effect of *CBL* and *SH2B3* mutations on the endothelial cells. However, further studies are needed to understand the exact contribution of the immune system, endothelium, and coagulation system in the biology of vascular damage in CBL syndrome.

This report presents some limitations. Experimental data are needed to define the exact biological mechanisms underpinning the relationship between *CBL* and immunological and coagulative disorders. Possible perturbations in PI3K/AKT/mTOR pathway need to be investigated in CBL syndrome [49–51]. Moreover, the role of adaptor proteins, like SH2B3, in the pathogenesis of immunological manifestations, necessarily requires further investigations.

Our data support the requirement of an extensive immunological characterization and monitoring of patients with CBL syndrome. Following confirmation of these findings, guidelines for surveillance and treatment of immune-related complications should be implemented. The present report also highlights the need for further investigation on coagulative and vascular alterations associated with CBL syndrome. Our findings suggest a cooperative role of pathogenic *SH2B3* and *CBL* variants in the development of immune dysregulation, also corroborated by the experimental evidence of the role of both these proteins in hematopoietic stem and immune system cells [40, 52]. In conclusion, this report contributes to expand the clinical spectrum of CBL syndrome, uncovering previously undescribed immunological and coagulative features to be taken into account for a more effective management, follow-up and treatment of this disorder.

## Supplementary Information

**Additional file 1: Supplementary Figure S1.** Photographs of patient III-3; **Supplementary Figure S2.** Bone marrow aspirate smear from patient III.2; **Supplementary Figure S3.** Sanger sequencing validations of CBL c.1141T>C (A) and SH2B3 c.1697G>A (B) mutations performed on peripheral blood cells of II.2 and III.2; **Supplementary Table S1.** Immunoglobulin (Ig) levels and antibody responses.

## Data Availability

The datasets used and analyzed during the current study available from the corresponding author on reasonable request.
