# Supplementary material for: Immune dysregulation associated with co-occurring germline CBL and SH2B3 variants
Source: Hum Genomics. 2022 Sep 19;16:40. doi: 10.1186/s40246-022-00414-y (PMC9484243; doi:10.1186/s40246-022-00414-y)
Supplement: Supplementary file 1 — Additional file 1: Supplementary Figure S1. Photographs of patient III-3; Supplementary Figure S2. Bone marrow aspirate smear from patient III.2; Supplementary Figure S3. Sanger sequencing validations of CBL c.1141T>C (A) and SH2B3 c.1697G>A (B) mutations performed on peripheral blood cells of II.2 and III.2; Supplementary Table S1. Immunoglobulin (Ig) levels and antibody responses. [file 40246_2022_414_MOESM1_ESM.pdf]

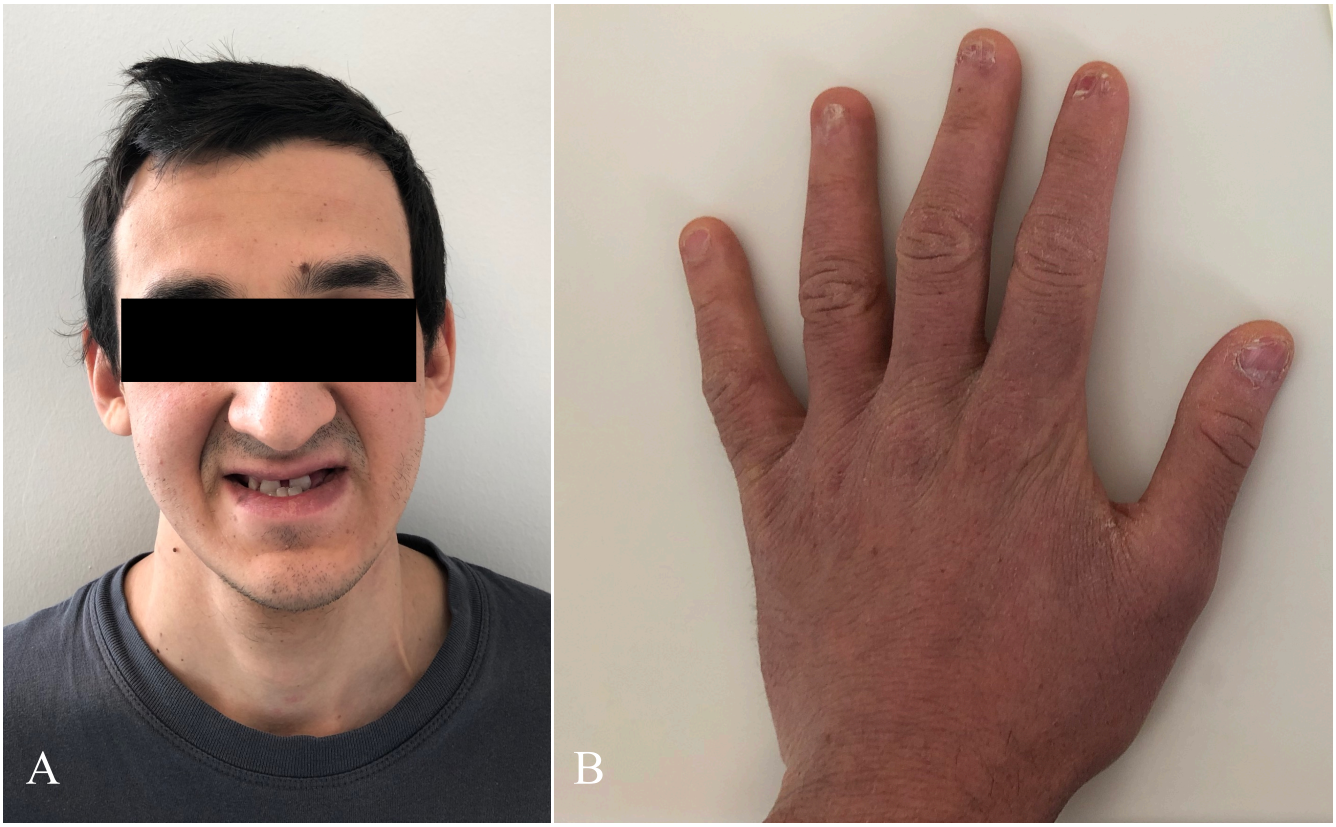

Supplementary Figure S1: Photographs of patient III-3. (A) Patient exhibits bulbous large nasal tip, fleshy and large ears, downslasting palpebral fissures, dental agenesia and onychodystrophy (B).

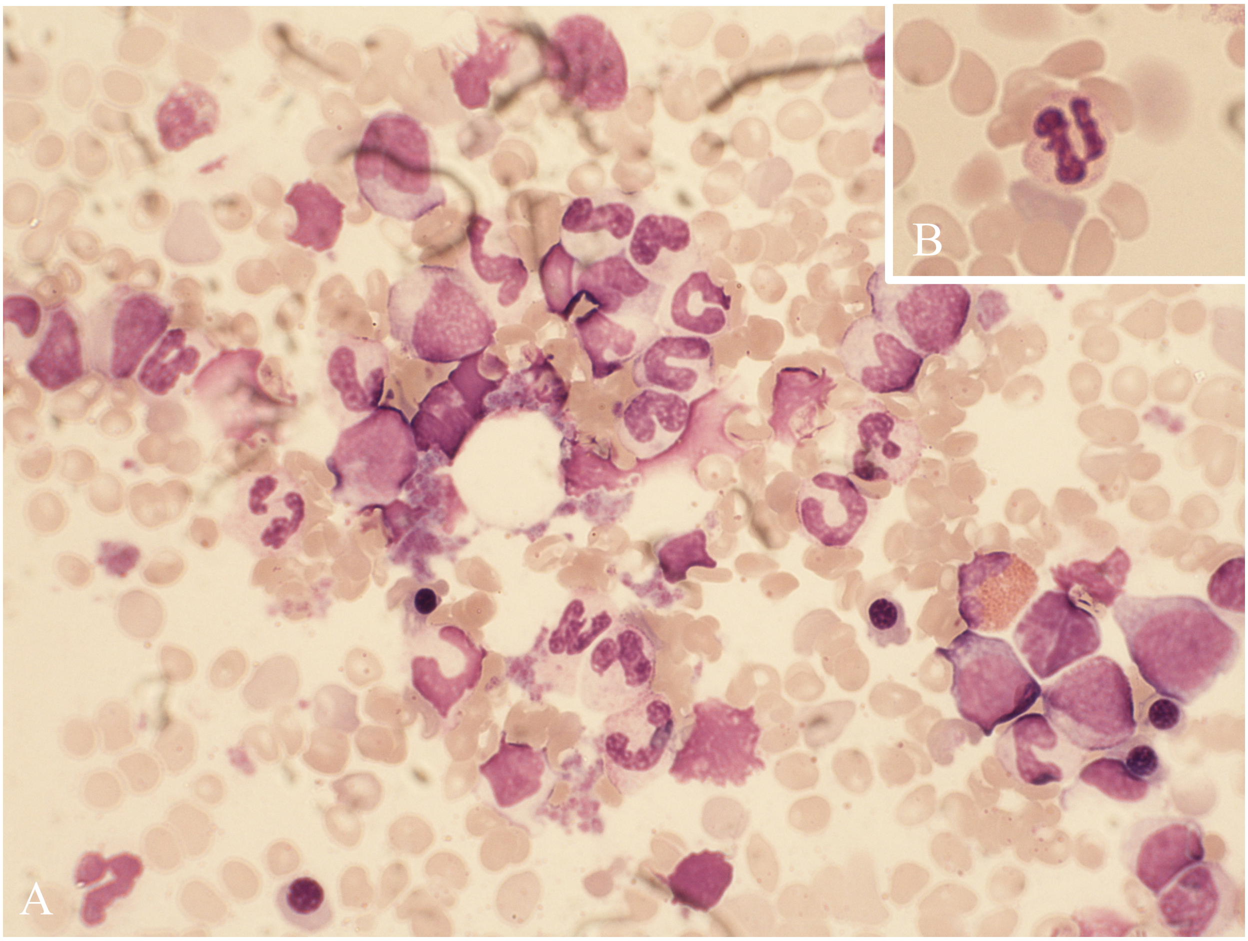

Supplementary Figure S2. Bone marrow aspirate smear from patient III.2. (A) Bone marrow aspirate film displays granulocytic hyperplasia with mild-to-moderate left shift in myeloid maturation in the absence of dysmorphic features. (B) Pelgeroid granulocyte.

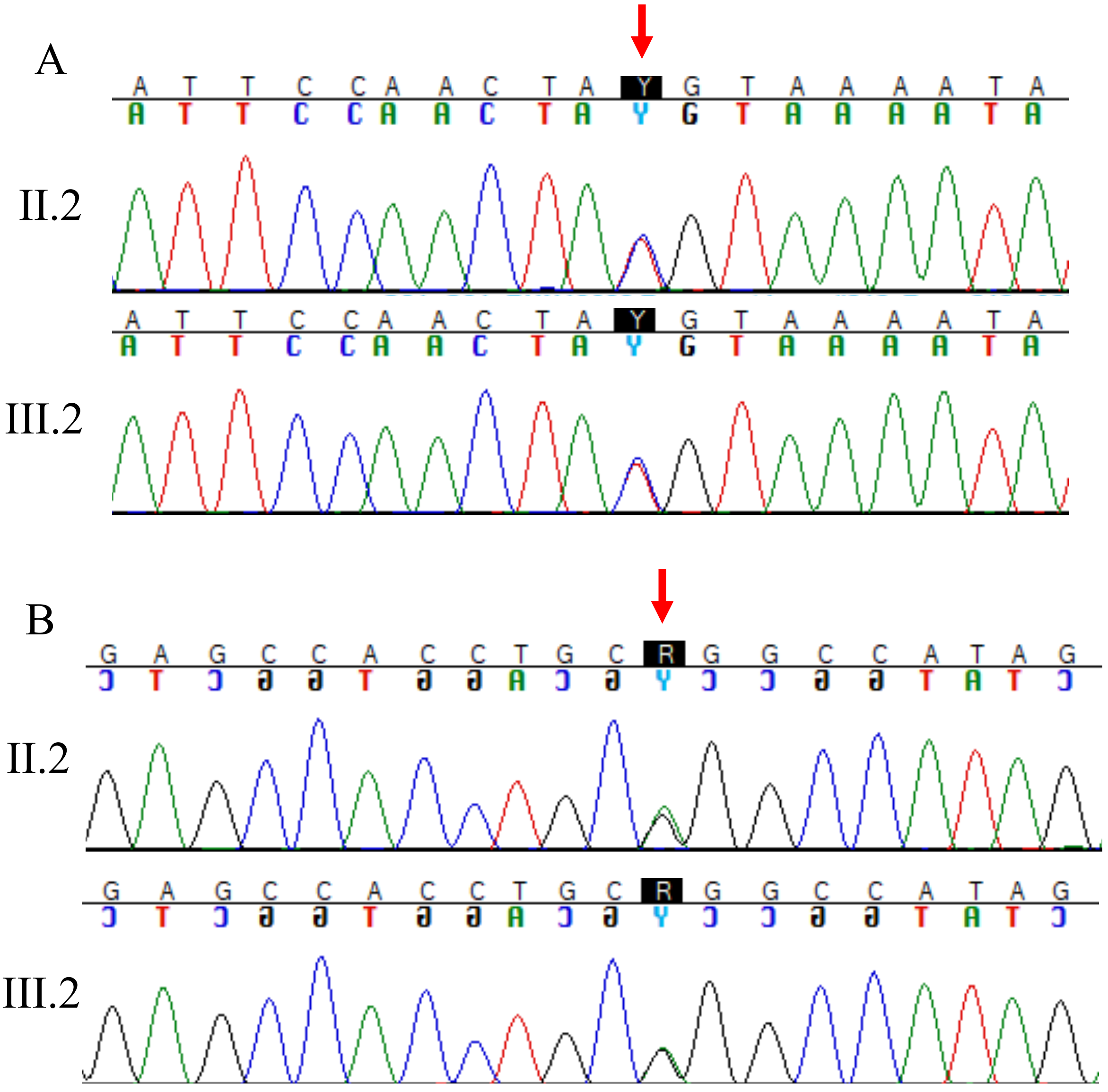

Supplementary Figure S3. Sanger sequencing validations of CBL c.1141T>C (A) and SH2B3 c.1697G>A (B) mutations performed on peripheral blood cells of II.2 and III.2.

	II-1	**II-2**	III-1	**III-2**	**III-3** (I-135)	*Normal Values for age (>16 yrs)*
IgG mg/dl [700-1600]	1255	907	1114	1129	947	700-1200
IgM mg/dl [40-230]	53	212	166	50	105	40-230
IgA mg/dl [70-400]	218	55	266	243	173	70-400
IgE UI/ml [<100]	n.d.	n.d.	23	271	155	<100
IgG1 mg/dl [382.4-928.6]	702.4	n.d.	550.5	708.1	512.3	382.4-928.6
IgG2 mg/dl [241.8-700.3]	561.7	n.d.	594.2	438.1	412.8	241.8-700-3
IgG3 mg/dl [21.8-176.1]	136.1	n.d.	70.5	83.7	107.5	21.8-176.1
IgG4 mg/dl [3.9-86.4]	36	n.d.	48.1	33.5	31.3	3.9-86.4
Measles (IgG) CLIA	Pos	n.d.	Neg	Pos	Pos	
Parotytis IgG) CLIA	Pos	n.d.	Neg	Pos	Pos	
Rubella (IgG) CLIA	Pos	n.d.	Pos	Pos	Pos	
Pertussis (IgG) EIA UI/ml	37	n.d.	12	18	20	> 7 protection
HBV (Ab. Anti HBs) ECLIA mUI/ml	2	n.d.	20	42	93	> 10 protection
Difterytis (IgG) EIA UI/ml	0.79	n.d.	0.12	0.05	1.14	> 0.1 protection
Tetanus (IgG) EIA UI/ml	4.79	n.d.	1.83	1.10	4.53	>0.5 protection

Supplementary Table S1. Immunoglobulin (Ig) levels and antibody responses. Results were within normal limits for age.
